# New diagnostic criteria for gestational diabetes mellitus and their impact on the number of diagnoses and pregnancy outcomes

**DOI:** 10.1007/s00125-017-4506-x

**Published:** 2017-11-22

**Authors:** Sarah H. Koning, Jelmer J. van Zanden, Klaas Hoogenberg, Helen L. Lutgers, Alberdina W. Klomp, Fleurisca J. Korteweg, Aren J. van Loon, Bruce H. R. Wolffenbuttel, Paul P. van den Berg

**Affiliations:** 1 Department of Endocrinology, University of Groningen, University Medical Center Groningen, HPC AA31, P.O. Box 30.001, Hanzeplein 1, 9700 RB Groningen, the Netherlands; 2 Laboratory of Clinical Chemistry, Certe, Medical Laboratory North, Groningen, the Netherlands; 3 Department of Internal Medicine, Martini Hospital, Groningen, the Netherlands; 4 Department of Internal Medicine, Medical Center Leeuwarden, Leeuwarden, the Netherlands; 5 Department of Obstetrics and Gynaecology, Martini Hospital, Groningen, the Netherlands; 6 Department of Obstetrics and Gynaecology, University of Groningen, University Medical Center Groningen, Groningen, the Netherlands

**Keywords:** Diagnosis, Diagnostic criteria, GDM, Gestational diabetes mellitus, Pregnancy, Pregnancy outcomes, WHO

## Abstract

**Aims/hypothesis:**

Detection and management of gestational diabetes mellitus (GDM) are crucial to reduce the risk of pregnancy-related complications for both mother and child. In 2013, the WHO adopted new diagnostic criteria for GDM to improve pregnancy outcomes. However, the evidence supporting these criteria is limited. Consequently, these new criteria have not yet been endorsed in the Netherlands. The aim of this study was to determine the impact of these criteria on the number of GDM diagnoses and pregnancy outcomes.

**Methods:**

Data were available from 10,642 women who underwent a 75 g OGTT because of risk factors or signs suggestive of GDM. Women were treated if diagnosed with GDM according to the WHO 1999 criteria. Data on pregnancy outcomes were obtained from extensive chart reviews from 4,431 women and were compared between women with normal glucose tolerance (NGT) and women classified into the following groups: (1) GDM according to WHO 1999 criteria; (2) GDM according to WHO 2013 criteria; (3) GDM according to WHO 2013 fasting glucose threshold, but not WHO 1999 criteria; and (4) GDM according to WHO 1999 2 h plasma glucose threshold (2HG), but not WHO 2013 criteria.

**Results:**

Applying the new WHO 2013 criteria would have increased the number of diagnoses by 45% (32% vs 22%) in this population of women at higher risk for GDM. In comparison with women with NGT, women classified as having GDM based only on the WHO 2013 threshold for fasting glucose, who were not treated for GDM, were more likely to have been obese (46.1% vs 28.1%, *p* < 0.001) and hypertensive (3.3% vs 1.2%, *p* < 0.001) before pregnancy, and to have had higher rates of gestational hypertension (7.8% vs 4.9%, *p* = 0.003), planned Caesarean section (10.3% vs 6.5%, *p* = 0.001) and induction of labour (34.8% vs 28.0%, *p =* 0.001). In addition, their neonates were more likely to have had an Apgar score <7 at 5 min (4.4% vs 2.6%, *p* = 0.015) and to have been admitted to the Neonatology Department (15.0% vs 11.1%, *p* = 0.004). The number of large for gestational age (LGA) neonates was not significantly different between the two groups. Women potentially missed owing to the higher 2HG threshold set by WHO 2013 had similar pregnancy outcomes to women with NGT. These women were all treated for GDM with diet and 20.5% received additional insulin.

**Conclusions/interpretation:**

Applying the WHO 2013 criteria will have a major impact on the number of GDM diagnoses. Using the fasting glucose threshold set by WHO 2013 identifies a group of women with an increased risk of adverse outcomes compared with women with NGT. We therefore support the use of a lower fasting glucose threshold in the Dutch national guideline for GDM diagnosis. However, adopting the WHO 2013 criteria with a higher 2HG threshold would exclude women in whom treatment for GDM seems to be effective.

**Electronic supplementary material:**

The online version of this article (10.1007/s00125-017-4506-x) contains peer-reviewed but unedited supplementary material, which is available to authorised users.

## Introduction

Gestational diabetes mellitus (GDM) is associated with an increased risk of pregnancy-related complications for both mother and child [1, 2]. International guidelines recommend active screening for GDM since many of these risks can be reduced by its detection and management [3, 4]. However, these guidelines lack uniformity in terms of their diagnostic thresholds.

In 2010, the International Association of the Diabetes and Pregnancy Study Groups (IADPSG) proposed more stringent thresholds for diagnosing GDM that were based on the results of the international prospective Hyperglycaemia and Adverse Pregnancy Outcomes (HAPO) study [5, 6]. This study demonstrated a linear association between maternal glucose levels at fasting and during an OGTT and the risk of adverse pregnancy outcomes such as increased birthweight, primary Caesarean section and neonatal hypoglycaemia [6]. The IADPSG diagnostic criteria (fasting plasma glucose ≥5.1 mmol/l; and/or 1 h plasma glucose ≥10.0 mmol/l; and/or 2 h plasma glucose (2HG) ≥8.5 mmol/l) have now been adopted by many guideline committees and expert groups, including the WHO in 2013 [5, 7].

However, evidence in support of applying the new criteria to diagnose GDM to improve pregnancy outcomes is limited. The optimal glucose thresholds to define GDM remain uncertain and international consensus has not yet been reached [8, 9]. Applying the new criteria gives rise to more women being diagnosed with GDM and the resulting cost increases and medicalisation of pregnancy are causes for concern for healthcare managers and caregivers [10, 11]. Studies into clinical outcomes and cost-effectiveness analyses are required to better appraise the value of these new glucose thresholds. In the Netherlands, the new WHO 2013 criteria have not yet been endorsed. In their 2010 guideline ‘Diabetes and Pregnancy’, the Dutch Society of Obstetrics and Gynaecology instead recommends using the WHO 1999 criteria to diagnose GDM (fasting glucose ≥7.0 mmol/l and/or 2HG ≥7.8 mmol/l) [12, 13]. When compared with the new WHO 2013 criteria, these use a much higher threshold for fasting glucose and a lower threshold for 2HG.

The consequences of adopting the WHO 2013 thresholds need to be evaluated in order to answer the following crucial questions: Do the additional women diagnosed with GDM using the new WHO 2013 fasting glucose criteria (fasting glucose ≥5.1 but ≤6.9 mmol/l) indeed have unfavourable pregnancy outcomes? What are the pregnancy outcomes of the women who would be missed owing to the higher 2HG threshold using the WHO 2013 criteria (i.e. women with 2HG ≥7.8 but ≤8.4 mmol/l)?

The aim of this study was therefore to evaluate the possible impact on the number of GDM diagnoses and pregnancy outcomes when applying the new WHO 2013 criteria instead of the older WHO 1999 criteria.

## Methods

### Study design and population

This study is a retrospective evaluation of data on testing for GDM (in women with relevant risk factors), pregnancy management and pregnancy outcomes collected between January 2011 and September 2016 in the Groningen area by Certe; a regional primary- and secondary healthcare laboratory in the north of the Netherlands and by the University Medical Center Groningen (UMCG); a tertiary referral centre.

As previously described [14, 15], pregnant women between 24 and 28 weeks of gestation were referred either by their midwife (in primary care) or by their gynaecologist (in secondary/tertiary care) for a 75 g OGTT if they had one or more risk factors for GDM [13]. These risk factors were; a pre-pregnancy body mass index (BMI) ≥30 kg/m^2^; having a first-degree relative with diabetes; having a previous neonate weighing ≥4500 g at birth or a birthweight >95th percentile; having a personal history of GDM, intrauterine fetal death or polycystic ovary syndrome; and belonging to an ethnic risk group (Asian, African-Caribbean, Middle Eastern i.e. Moroccan and Egyptian). Universal testing is not recommended in the Dutch national guideline.

An OGTT was also recommended for women with signs suggestive of GDM (e.g. fetal macrosomia or polyhydramnios). Women were treated if diagnosed with GDM according to the WHO 1999 criteria: fasting glucose ≥7.0 mmol/l and/or 2HG value ≥7.8 mmol/l [12]. All women were referred to a dietitian for dietary counselling and received instructions for self-monitoring of blood glucose values by a diabetes specialist nurse. If, after 1–2 weeks, repeated measurements indicated a fasting glucose level >5.3 mmol/l and/or 1 h postprandial plasma glucose level >7.8 mmol/l, insulin therapy was started [15].

The study was conducted in accordance with the guidelines of the Declaration of Helsinki and Good Clinical Practice, and approved by the Medical Ethical Review Committee of the UMCG. The data was analysed retrospectively and all requirements for patient anonymity are in agreement with the regulations of the ethical committee of both hospitals for publication of patient data. According to this and the Dutch law Medical Research with Human Subject, no informed consent was deemed necessary.

### GDM classification and outcomes

On the basis of their OGTT results, women were retrospectively assigned to the following diagnostic groups:

1. normal glucose tolerance (fasting glucose <5.1 mmol/l and 2HG <7.8 mmol/l), denoted as ‘NGT’;
2. GDM according to WHO 1999 criteria (fasting glucose ≥7.0 mmol/l and/or 2HG ≥7.8 mmol/l), denoted as ‘WHO 1999’;
3. GDM according to WHO 2013 criteria (fasting glucose ≥5.1 mmol/l and/or 2HG ≥8.5 mmol/l), denoted as ‘WHO 2013’.

We separately identified two further groups of women as follows:

4. GDM according to new WHO 2013 fasting glucose threshold, but do not meet WHO 1999 criteria (fasting glucose ≥5.1 but ≤6.9 mmol/l and 2HG <7.8 mmol/l), denoted as ‘WHO 2013 only fasting glucose’;
5. GDM according to WHO 1999 2HG threshold, but do not meet WHO 2013 criteria (fasting glucose <5.1 mmol/l and 2HG ≥7.8 but ≤8.4 mmol/l), denoted as ‘WHO 1999 only 2HG’.

It should be noted that the women with NGT underwent an OGTT because they had risk factors for GDM or signs suggestive of GDM (e.g. fetal macrosomia or polyhydramnios). Approximately 85% of the women were tested based on predefined risk factors for GDM. Since the women with NGT are not representative of all pregnancies not affected by GDM, neonatal outcomes regarding birthweight in the general obstetric population in the Northern region of the Netherlands (period 2011–2013) were obtained from the Dutch Perinatal Registry and the Municipal Health Service Groningen. The nature of this dataset unfortunately does not allow exclusion of those screened for GDM.

**Fig. 1:**
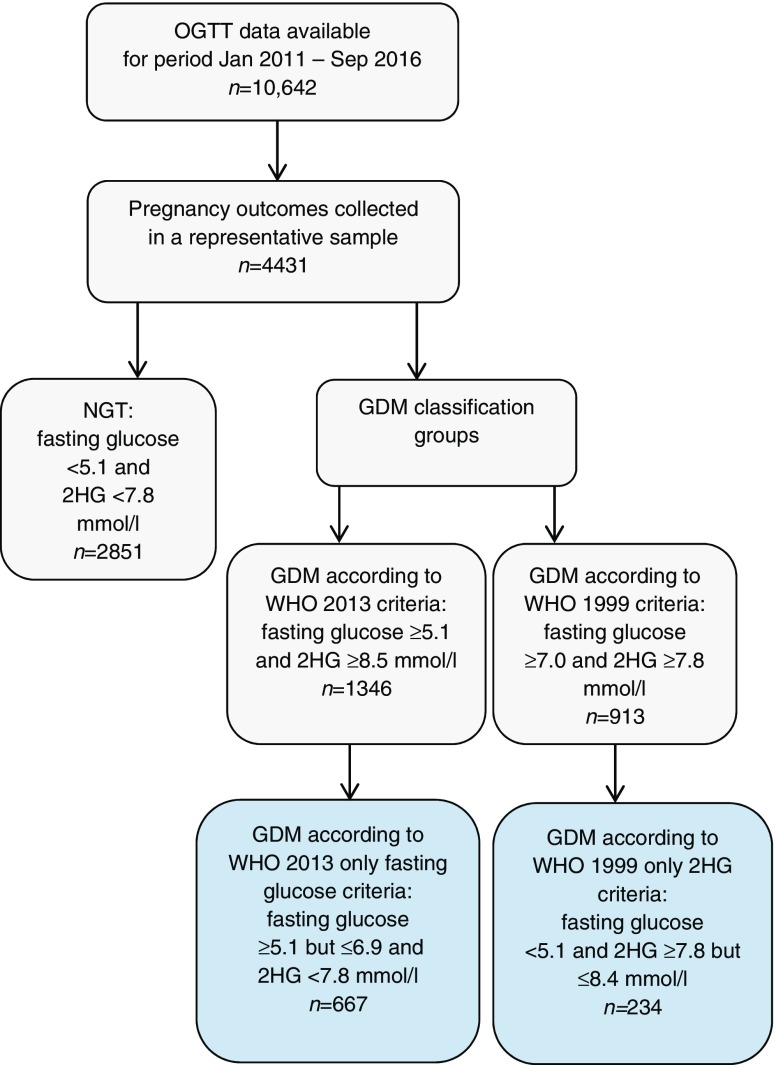
Flow chart of the study population

The original OGTT dataset comprised 10,642 women with GDM risk factors or signs suggestive of GDM. We were able to retrospectively collect data on maternal characteristics and pregnancy outcomes in a representative sample of women (*n* = 4431) from written medical and obstetric records at midwives’ offices in primary care and at two hospitals; the UMCG and the Martini Hospital Groningen (Fig. 1). All data were incorporated in an anonymised database. An overview of collected maternal and neonatal outcomes is given in ESM Table 1.

### Statistical analyses

Continuous data are presented as mean ± SD, or as median and interquartile range (IQR) in case of skewed distribution. Categorical data are presented as numbers and percentages. Differences between groups were tested using the Student’s unpaired *t* test for continuous data, or the Mann–Whitney *U* test in case of skewed distribution. For categorical data, a *χ* ^2^ test or Fisher’s exact test was used.

All *p* values are two-tailed, and *p* values <0.05 are considered statistically significant.

## Results

### Number of GDM diagnoses and maternal characteristics

OGTT data were collected from 10,642 pregnant women with GDM risk factors or signs suggestive of GDM. In Table 1, numbers of women with GDM are presented according to the WHO 1999 criteria and WHO 2013 criteria. The number of women with GDM in the total cohort was 22% when the WHO 1999 criteria were applied and 32% when the WHO 2013 criteria were applied.

**Table 1:** Number of GDM diagnoses according to the WHO 1999 and WHO 2013 criteria

Criteria mmol/l	WHO 1999 Fasting glucose ≥7.0 and/or 2HG ≥7.8	WHO 2013 Fasting glucose ≥5.1 and/or 2HG ≥8.5
Total cohort *n* = 10,642		
Total GDM, *n* (%)	2326 (22)	3364 (32)
GDM based on elevated fasting glucose, but with 2HG below the threshold, *n* (%)	14 (1)	2045 (61)
GDM based on the 2HG, but with fasting glucose below the threshold, *n* (%)	2267 (97)	634 (19)
GDM based on both elevated fasting glucose and elevated 2HG, *n* (%)	45 (2)	685 (20)

Abbreviations: 2HG, 2 h plasma glucose

The characteristics of the women in the different GDM classification groups are presented in Table 2. Characteristics and pregnancy outcomes were collected for 4,431 women who had singleton pregnancies. The fasting glucose, 2HG values and age of these 4,431 women (fasting glucose 4.7 ± 0.6 mmol/l; 2HG 6.6 ± 1.6 mmol/l; age of the mother at OGTT 30.6 ± 4.9 years) were similar to the values obtained for the other 6,211 women (mean fasting glucose 4.8 ± 0.5 mmol/l; 2HG 6.6 ± 1.6 mmol/l; age of the mother at OGTT 30.1 ± 4.9 years) who completed a 75 g OGTT. Treatment for GDM was only given to women diagnosed according to the WHO 1999 criteria, since the WHO 2013-based GDM classification was only assigned retrospectively. In comparison with women in the NGT group, women classified as having GDM (using the WHO 2013 criteria or WHO 1999 criteria) were older, had a higher pre-pregnancy BMI and were more likely to be multiparous and to have chronic hypertension.

**Table 2:** Maternal characteristics according to the GDM classification groups

		NGT	WHO 1999	WHO 2013	WHO 2013 only fasting glucose	WHO 1999 only 2HG
	Criteria (mmol/l)	Fasting glucose <5.1 and 2HG <7.8	Fasting glucose ≥7.0 and/or 2HG ≥7.8	Fasting glucose ≥5.1 and/or 2HG ≥8.5	Fasting glucose ≥5.1-≤6.9 and 2HG <7.8	Fasting glucose <5.1 and 2HG ≥7.8-≤8.4
Characteristics	*N* ^a^					
*N*	4431	2851	913	1346	667	234
Treated for GDM, *n* (%) Diet		0	524 (57.4)	338 (25.1)	0	186 (79.5)
Additional insulin therapy		0	389 (42.6)	341 (25.3)	0	48 (20.5)
Age (years)	4431	30.7 ± 4.9	32.1 ± 5.1***	32.0 ± 5.2***	31.6 ± 5.2***	31.6 ± 4.5**
Pre-pregnancy BMI (kg/m^2^)	4196	25.2 (22.0–30.4)	27.7 (24.1–31.8)***	28.7 (24.5–32.9)***	29.1 (24.8–33.5)***	26.4 (23.3–30.4)**
Pre-pregnancy BMI, *n* (%)^b^	4196		***	***	***	***
<25 kg/m^2^		1311 (48.8)	285 (32.0)	366 (28.5)	167 (26.9)	86 (37.4)
25–30 kg/m^2^		618 (23.0)	276 (30.9)	365 (28.4)	167 (26.9)	78 (33.9)
≥30 kg/m^2^		755 (28.1)	331 (37.1)	551 (42.9)	286 (46.1)	66 (28.7)
Ethnicity, *n* (%)^b^	4431		*		*	*
White		2211 (77.6)	719 (78.8)	1060 (78.8)	519 (77.8)	178 (76.1)
Asian		160 (5.6)	65 (7.1)	62 (4.6)	21 (3.1)	24 (10.3)
African-American		150 (5.3)	37 (4.1)	78 (5.8)	48 (7.2)	7 (3.0)
Mediterranean		207 (7.3)	68 (7.4)	95 (7.1)	47 (7.0)	20 (8.5)
Other		123 (4.3)	24 (2.6)	51 (3.8)	32 (4.8)	5 (2.1)
Nulliparous, *n* (%)	4431	1281 (44.9)	373 (40.9)*	523 (38.9)***	250 (37.5)***	100 (42.7)
Chronic hypertension, *n* (%)	4427	34 (1.2)	37 (4.1)***	52 (3.9)***	22 (3.3)***	7 (3.0)*
Smoking during pregnancy, *n* (%)	4381	296 (10.5)	101 (11.1)	165 (12.4)	87 (13.2)*	23 (9.8)

Data are expressed as mean ± SD, median (IQR) or proportion of *n* (%)

*p* values are based on Student’s unpaired *t* test (non-skewed continuous variables), Mann–Whitney *U* test (skewed continuous variables), or *χ* ^2^ test/Fisher’s exact test. *p<0.05, **p<0.01, ***p<0.001 compared with NGT group

^a^Data with respect to pre-pregnancy BMI, chronic hypertension and smoking are missing in 235 (5.3%), 4 (0.09%) and 50 (1.1%) of the subjects, respectively

^b^ *p* values are for the complete categorical variable BMI and ethnicity, indicating changes in ethnic composition and BMI composition between groups

A total of 667 women were retrospectively classified as having GDM based only on the WHO 2013 fasting glucose criteria. In comparison with women in the NGT group, these women were older, had a higher pre-pregnancy BMI (29.1 [IQR 24.8–33.5] vs 25.2 [IQR 22.0–30.4] kg/m^2^, *p* < 0.001), were more likely to be obese (46.1% vs 28.1%, *p* < 0.001), to have smoked during pregnancy (13.2% vs 10.5%, *p* = 0.05) and to have chronic hypertension (3.3% vs 1.2%, *p* < 0.001).

A total of 234 women were retrospectively classified as having GDM based only on the WHO 1999 criteria for 2HG. These women were all treated for GDM, 79.5% with diet only and 20.5% received additional insulin therapy. In comparison with women with NGT, women in this group were older, had a slightly higher pre-pregnancy BMI (26.4 [IQR 23.3–30.4] vs 25.2 [IQR 22.0–30.4] kg/m^2^, *p* = 0.01), were more likely to be overweight (33.9% vs 23.0%, *p* < 0.001) and to have hypertension (3.0% vs 1.2%, *p* = 0.02).

### Pregnancy outcomes

Maternal and neonatal outcomes according to the different GDM classification groups are given in Table 3. Compared with women in the NGT group, women classified as having GDM (using the WHO 2013 criteria or WHO 1999 criteria) were more likely to develop gestational hypertension or preeclampsia and to have had a planned Caesarean section delivery or induced labour.

**Table 3:** Pregnancy outcomes according to the GDM classification groups

			NGT	WHO 1999	WHO 2013	WHO 2013 fasting glucose only	WHO 1999 2HG only
Pregnancy outcomes	General obstetric population in the north of the Netherlands	Criteria (mmol/l)	Fasting glucose <5.1 and 2HG <7.8	Fasting glucose ≥7.0 and/or 2HG ≥7.8	Fasting glucose ≥5.1 and/or 2HG ≥8.5	Fasting glucose ≥5.1- ≤ 6.9 and 2HG <7.8	Fasting glucose <5.1 and 2HG ≥7.8- ≤ 8.4
		*N* ^b^					
*N*	29,562	4431	2851	913	1346	667	234
Treated for GDM, *n*			0	913	679	0	234
Maternal							
Gestational hypertension, *n* (%)		4427	139 (4.9)	62 (6.8)*	98 (7.3)**	52 (7.8)**	16 (6.9)
Preeclampsia, *n* (%)		4427	41 (1.4)	28 (3.1)**	35 (2.6)**	12 (1.8)	5 (2.1)
Induction of labour, *n* (%)		4405	793 (28.0)	587 (64.3)***	670 (50.0)***	230 (34.8)**	147 (62.8)***
Mode of delivery, *n* (%)		4410					
Vaginal			2051 (72.3)	618 (67.7)**	904 (67.4)**	451 (68.1)*	165 (70.5)
Emergency CS			327 (11.5)	116 (12.7)	177 (13.2)	89 (13.4)	28 (12.0)
Planned CS			185 (6.5)	103 (11.3)***	150 (11.2)***	68 (10.3)**	21 (9.0)
Instrumental			272 (9.6)	76 (8.3)	110 (8.2)	54 (8.2)	20 (8.5)
Gestational age at delivery (weeks)		4431	39.7 (38.7–40.6)	38.3 (38.0–39.0)***	38.7 (38.0–39.9)***	39.6 (38.3–40.4)***	38.6 (38.1–39.4)***
Neonatal							
LGA, *n* (%)	3246 (11.0)	4430	514 (18.0)	167 (18.3)	271 (20.1)	140 (21.0)	36 (15.4)
Macrosomia, *n* (%)	4275 (14.5)	4431	595 (20.9)	108 (11.8)***	226 (16.8)**	148 (22.2)	30 (12.8)**
Small for gestational age, *n* (%)	2364 (8.0)	4430	195 (6.8)	36 (3.9)**	69 (5.1)*	38 (5.7)	5 (2.1)**
Birthweight (g)		4431	3544 ± 579	3391 ± 550***	3477 ± 590**	3580 ± 596	3437 ± 498**
Birth trauma, *n* (%)		4420	64 (2.3)	27 (3.0)	43 (3.2)	20 (3.0)	4 (1.7)
Hypoglycaemia, *n* (%)^a^		4418	NA	38 (4.2)***	NA	NA	4 (1.7)
Hyperbilirubinaemia, *n* (%)^a^		4418	NA	24 (2.6)**	NA	NA	5 (2.1)
Stillbirth, *n* (%)		4431	10 (0.4)	2 (0.2)	6 (0.4)	4 (0.6)	0
Preterm delivery, *n* (%)		4431	146 (5.1)	57 (6.2)	92 (6.8)*	46 (6.9)	11 (4.7)
Respiratory support, *n* (%)		4418	116 (4.1)	34 (3.7)	51 (3.8)	27 (4.1)	10 (4.3)
Apgar score <7 at 5 min, *n* (%)		4414	74 (2.6)	30 (3.3)	57 (4.3)**	29 (4.4)*	2 (0.9)
Admission to neonatology, *n* (%)		4423	315 (11.1)	130 (14.2)*	206 (15.3)***	100 (15.0)**	24 (10.3)

Data are expressed as mean ± SD, median (IQR) or proportion of *n* (%)

^a^Data were collected in primary care (midwives) and secondary care (hospital). In primary care, neonatal hypoglycaemia and hyperbilirubinaemia were not reported and measured in all pregnancies. Therefore we only report the percentages for the WHO 1999 group, as all these women delivered in secondary care^b^ Data with respect to gestational hypertension, preeclampsia, induction of labour, mode of delivery, birth trauma, hypoglycaemia, hyperbilirubinaemia, respiratory support, Apgar score and admission to the neonatology are missing in 4 (0.09%), 4 (0.09%), 26 (0.5%), 21 (0.5%), 11 (0.2%), 13 (0.3%), 13 (0.3%), 13 (0.3%), 17 (0.4%) and 8 (0.2%) of the subjects, respectively

*p* values are based on Student’s unpaired *t* test (non-skewed continuous variables), Mann–Whitney *U* test (skewed continuous variables), or *χ* ^2^ test/Fisher’s exact test. *p<0.05, **p<0.01, ***p<0.001 compared with NGT group

Compared with women in the NGT group, women classified as having GDM based only on the WHO 2013 criteria for fasting glucose were more likely to have gestational hypertension (7.8% vs 4.9%, *p* = 0.003), to have a planned Caesarean section (10.3% vs 6.5%, *p* = 0.001) and induced labour (34.8% vs 28.0%, *p* = 0.001).

Women classified as having GDM based only on the WHO 1999 criteria for 2HG were more likely to have induced labour (62.8% vs 28.0%, *p* < 0.001) compared with women in the NGT group. There were no significant differences in gestational hypertension, preeclampsia or mode of delivery between this group and women with NGT.

Neonates from mothers classified as having GDM (using the WHO 2013 criteria or WHO 1999 criteria) had a lower birthweight, a lower gestational age at delivery and were less likely to have macrosomia compared with those from mothers with NGT. However, the likelihood of these neonates being born large for gestational age (LGA) [16] did not differ significantly from that of neonates born to women with NGT. The likelihood of these neonates being born small for gestational age (SGA) [16] was lower than that of neonates born to women with NGT.

Compared with neonates from mothers with NGT, neonates from mothers classified as having GDM based only on the WHO 2013 criteria for fasting glucose had similar birthweight (3580 ± 596 g vs 3544 ± 579 g, *p* = 0.145), likelihood of having macrosomia (22.2% vs 20.9%, *p* = 0.452) or being born LGA (21.0% vs 18.0%, *p* = 0.077). However, these neonates were more likely to have had an Apgar score <7 after 5 min (4.4% vs 2.6%, *p* = 0.015) and to have been admitted to the neonatology department (15.0% vs 11.0%, *p* = 0.004). None of the other neonatal outcomes showed significant differences between these two groups.

Compared with neonates from mothers with NGT, neonates from mothers classified as having GDM based only on the WHO 1999 criteria for 2HG had a lower birthweight (3437 ± 498 g vs 3544 ± 579 g, *p* = 0.01) and were less likely to have macrosomia (12.8% vs 20.9%, *p* = 0.003). The likelihood of these neonates being born LGA was similar to those from mothers with NGT (15.4% vs 18.0%, *p* = 0.309). However, 20.5% of the women in this group were treated with insulin. None of the other neonatal outcomes showed significant differences between these two groups.

When we compared the percentage of LGA neonates in our data with those found in the general obstetric population in the north of the Netherlands (11%), we found that all GDM classification groups as well as women with NGT had a higher percentage of LGA neonates.

## Discussion

This large retrospective cohort study to evaluate the possible impact of applying the new WHO 2013 criteria demonstrates that the number of GDM diagnoses would increase by 45%, relative to the WHO 1999 criteria. We also show that applying these new criteria indeed identifies a new group of women (with fasting glucose ≥5.1 but ≤6.9 mmol/l) who have unfavourable characteristics and more adverse pregnancy outcomes when compared either with women found to have NGT upon testing or with the general obstetric population. Our results show that women potentially missed owing to the higher 2HG threshold (2HG ≥7.8 but ≤8.4 mmol/l) of the WHO 2013 criteria have similar pregnancy outcomes to women with NGT. Our results also indicate that neonates from mothers who are tested for GDM but are found to have NGT are more likely to be born LGA or with macrosomia than those born to mothers in the general obstetric population in our region.

### The number of gestational diabetes diagnoses and maternal characteristics

Several authors have expressed concerns about the adoption of the WHO 2013 criteria, as this will significantly increase the number of GDM diagnoses, and impose a higher burden on healthcare provided by obstetricians [10, 11]. Studies have shown that implementing the new WHO 2013 criteria will result in a two- to threefold increase in the number of GDM diagnoses [9, 11, 17]. The increase from 22% to 32% observed in our cohort of women at higher risk of GDM was mainly the result of an increase in the number of women who would be diagnosed on the basis of an elevated fasting glucose level. At the same time a number of women would not be diagnosed due to the higher threshold for 2HG in the WHO 2013 criteria.

The lower fasting glucose threshold in the WHO 2013 criteria identifies a group of women who are more likely than those with NGT to be obese and hypertensive. Moreover, the women classified as having GDM based only on the WHO 1999 criteria for 2HG were also more likely than women with NGT to be overweight. It is known that impaired fasting glucose (IFG; fasting glucose ≥5.6 and ≤6.9 mmol/l) and/or impaired glucose tolerance (IGT; 2HG ≥7.8 and ≤11.0 mmol/l) are both predictors for the future development of type 2 diabetes [18]. IFG and IGT are associated with an unfavourable metabolic profile, including obesity and hypertension. Both groups successfully identify a group of high-risk women with an adverse metabolic profile. These women are at increased risk of developing complications during pregnancy.

### Pregnancy outcomes

Although uncertainty remains regarding the optimal glucose threshold to define GDM, hyperglycaemia during pregnancy is clearly associated with an increased risk of adverse pregnancy outcomes [6]. Indeed, women in this study classified as having GDM using any criteria had higher rates of adverse maternal outcomes, including hypertensive disorders during pregnancy, planned Caesarean section and induced labour when compared with women with NGT. Moreover, the neonates of mothers classified as having GDM by any criteria were likely to have been admitted to the neonatology department.

Concerns have been raised about the ‘medicalisation’ of pregnancy should the new WHO 2013 criteria be implemented [10]. Based on the WHO 1999 criteria currently applied in the Netherlands, these women are not diagnosed with GDM and are therefore not treated with diet and/or insulin. However, our findings suggest that these women already have higher intervention rates. We demonstrated that, in contrast to women with NGT, women classified as having GDM based only on the WHO 2013 criteria for fasting glucose had higher rates of gestational hypertension, planned Caesarean section and induced labour and their neonates were more likely to have an Apgar score <7 at 5 min and to be admitted to the neonatology department. A number of other studies have also shown that women reclassified as having gestational diabetes using the new WHO 2013 criteria are at increased risk of adverse pregnancy outcomes including gestational hypertension, Caesarean section, neonatal intensive care admission and LGA neonates [19–22].

In terms of the likelihood of having an LGA neonate, we found no significant differences between the women classified as having GDM based on the WHO 2013 threshold for fasting glucose and women with NGT. However, the percentage of women in this group having an LGA neonate was much higher than for the general obstetric population (21% vs 11%). On the basis of these findings, it seems that women classified with GDM based only on the WHO 2013 criteria for fasting glucose should not be left untreated. This is supported by the results of a study by Landon et al, 2009, suggesting that early treatment in women with mild GDM reduces the percentage of women giving birth to LGA neonates by 7% [4].

Our study has also shown that implementing the new WHO 2013 criteria with a higher 2HG threshold may exclude a group of women who now benefit from treatment. The women classified as having GDM based only on the WHO 1999 criteria for 2HG had pregnancy outcomes similar to those of women with NGT. A notable finding was that they had the lowest rate of LGA neonates of all other diagnostic groups. The only obstetric variable that differed in comparison with the NGT group was the rate of induced labour, but it has to be borne in mind that induction of labour at 38/39 weeks of gestation is more likely to be recommended in women being treated for GDM, especially those receiving insulin therapy.

All women classified as having GDM based on the WHO 1999 criteria were actively treated with diet and/or insulin. These interventions normalised their glycaemic profile and outcomes for this group [14, 15]. Therefore, it is unclear whether these women can be safely left untreated after implementing the new WHO 2013 criteria. Indeed, Farrar et al, 2015, showed that even women with a 2HG glucose level ≥7.5 mmol/l are at increased risk of adverse outcomes (i.e. birthweight >90th percentile, high infant adiposity, and Caesarean section) [23]. These authors therefore recommend using a 2HG glucose threshold even lower than those recommended by both the WHO 1999 and WHO 2013 criteria.

A notable finding of our study is that the women who had undergone an OGTT and were subsequently found to have NGT also had a rate of LGA neonates higher than that of the women receiving treatment after being diagnosed with GDM based on the WHO 1999 criteria for 2HG (18.0% vs 15.4%). Although this finding was not statistically significant, it was a large difference compared with the incidence of LGA neonates in the general obstetric population (18% vs 11%). This coincidental finding shows that even NGT women without a positive diagnosis of GDM are at increased risk of giving birth to an LGA neonate. This finding is in agreement with a study by Meek et al, 2015, who demonstrated that women diagnosed and treated for GDM according to the National Institute for Health and Care Excellence (NICE) criteria in the UK had lower rates of LGA neonates than women negative for GDM according to both the NICE and IADPSG/WHO 2013 criteria [21]. A possible explanation for this finding is that these women were tested too early in pregnancy and were therefore not diagnosed with GDM at this time. Some studies have shown that the OGTT has a poor reproducibility, suggesting that some women who first test negative for GDM can test positive on a second test [24]. We therefore agree with the suggestion made by Meek et al, 2015, that standard lifestyle interventions (including dietary advice) given to women with GDM might also benefit NGT women.

### Strengths and limitations

A major strength of our study is the relatively large cohort of laboratory results from 75 g OGTTs and the extensive and detailed information regarding pregnancy outcomes in a subset of 4431 women with singleton pregnancies. All women with GDM were treated according to a detailed protocol in two large hospitals [14, 15]. Maternal and pregnancy outcome data were collected manually from individuals’ charts at their midwives’ offices. This study also has limitations that should be noted. First, since universal testing for GDM is not currently recommended in the Netherlands, only women with one or more risk factors for GDM or signs suggestive of GDM, such as macrosomia, were tested. The number of pregnancies affected by GDM found in our study is therefore not a reflection of the general obstetric population, and represents a selected group of women at higher risk of GDM. Universal testing is now recommended in several countries around the world. However, literature regarding the best method of screening (universal or risk-based) remains controversial [8]. Second, the WHO 2013 criteria also recommend that the diagnosis of GDM should include a 1 h plasma glucose of ≥10.0 mmol/l following a 75 g OGTT. Since we did not have data for 1 h glucose levels, the number of GDM diagnoses reported here might be an underestimation. Third, all women diagnosed according to the WHO 1999 criteria were offered treatment for GDM. Finally, we have compared outcomes with those in the general population in the north of the Netherlands between 2011 and 2013. Unfortunately data after 2013 have not yet been made available from public datasets. Furthermore, this dataset does not indicate which women were tested with an OGTT.

#### Conclusions

This large retrospective cohort study evaluated the possible impact on the number of GDM diagnoses and pregnancy outcomes of applying the new WHO 2013 rather than the old WHO 1999 criteria. We demonstrated that the number of GDM diagnoses would increase markedly if the WHO 2013 criteria were implemented. Nevertheless, the WHO 2013 threshold for fasting glucose, ≥5.1 but ≤6.9 mmol/l, identifies a group of women with an increased risk of pregnancy complications. We therefore recommend that, to improve GDM outcomes, the fasting glucose threshold in the Dutch national guideline needs to be reduced. However, it remains unclear from our data whether women with a 2HG ≥7.8 but ≤8.4 mmol/l can be safely left untreated. Recent studies suggest that a 2HG threshold of 7.5 mmol/l may be more appropriate. Future studies should evaluate whether a stricter 2HG OGTT threshold further improves pregnancy outcomes and should also address pregnancy outcomes in women who are tested but found to have NGT.

## Electronic supplementary material

ESM Table 1 (PDF 156 kb)

## Abbreviations

2HG: 2 h plasma glucose
GDM: Gestational diabetes mellitus
HAPO: Hyperglycaemia and Adverse Pregnancy Outcomes
IADPSG: International Association of the Diabetes and Pregnancy Study Groups
IFG: Impaired fasting glucose
IGT: Impaired glucose tolerance
IQR: Interquartile range
LGA: Large for gestational age
NGT: Normal glucose tolerance
NICE: National Institute for Health and Care Excellence
SGA: Small for gestational age
UMCG: University Medical Center Groningen
