# Supplementary material for: New diagnostic criteria for gestational diabetes mellitus and their impact on the number of diagnoses and pregnancy outcomes
Source: Diabetologia. 2017 Nov 22;61(4):800–9. doi: 10.1007/s00125-017-4506-x (PMC6449063; doi:10.1007/s00125-017-4506-x)
Supplement: Supplementary file 1 — (PDF 156 kb) [file 125_2017_4506_MOESM1_ESM.pdf]

ESM Table 1 Maternal and neonatal outcomes of interest

Outcomes	Definition
**Maternal**	
Gestational hypertension	A systolic blood pressure ≥140 mmHg and/or a diastolic blood pressure ≥90 mmHg, after 20 weeks of gestation in a previously normotensive woman
Preeclampsia	Gestational hypertension together with the presence of proteinuria (≥300 mg/24 hrs.) and also included women who had eclampsia and Hemolysis Elevated Liver enzymes and Low Platelets (HELLP) syndrome
Induction of labour	Process to start childbirth
Mode of delivery	Spontaneous vaginal delivery, instrumental delivery, emergency caesarean section, and planned caesarean section
Gestational age at delivery	The number of weeks that a baby has been in the uterus
**Neonatal**	
Birth weight	Body weight of the baby at birth (grams)
Neonate born large for gestational age (LGA)	Birth weight >90th percentile corrected for gestational age, sex, parity, and ethnic background
Macrosomia	Birth weight >4000 gram
Neonate born small for gestational age (SGA)	Birth weight <10th percentile corrected for gestational age, sex, parity, and ethnic background
Birth trauma	Shoulder dystocia, fracture of humerus or clavicle, brachial plexus injury
Hypoglycaemia	Occurring >2 hrs. after birth and defined as a having a blood glucose level <2.6 mmol/l or requiring treatment with glucose infusion [a]
Hyperbilirubinaemia	Requiring treatment with phototherapy after birth [a]
Stillbirth	Delivery of a baby showing no signs of life
Preterm delivery	Delivery <37 weeks of gestation
Requirements for respiratory support	The need to intubate or apply continuous positive airway pressure
Apgar score	Score <7 at 5 min
Admission to the Neonatology Department	NICU, Neonatal Intensive Care Unit

[a] Data were collected in primary care (midwives) and secondary care (hospital). In primary care, hypoglycaemia and hyperbilirubinaemia were not reported and measured in all neonates. Therefore we only report the percentages for the WHO-1999 group, as these women delivered in secondary care
